# Immune response after laparoscopic colectomy for cancer: a review

**DOI:** 10.1093/gastro/got014

**Published:** 2013-05-03

**Authors:** Styliani Karanika, Theodoros Karantanos, George E. Theodoropoulos

**Affiliations:** Colorectal Unit, First Department of Propaedeutic Surgery, Athens Medical School, Athens, Greece

**Keywords:** colorectal cancer, laparoscopic colectomy, immune response, innate immunity, cellular immunity

## Abstract

**Background and aim:** Colorectal cancer (CRC) is the third leading cause of cancer mortality worldwide and laparoscopic colectomy has been established as equivalent to the open approach in terms of oncological results and patients’ safety. Survival benefits have been reported in favor of laparoscopic colectomy (LC) in stage III CRC patients. Different immune responses after surgery, in terms of innate and cellular immunity, may potentially explain some of the reported differences. This review summarizes the literature on differences in immune response after the laparoscopic and the open approach for CRC.

**Materials and Methods:** A literature search of electronic databases was conducted and all studies published on ‘colorectal cancer’, ‘laparoscopic and open colectomy’ ‘immune response’ and ‘surgical stress laparoscopy versus open’ were collected. Among these, the ones referring to CRC and those that had any clinical relevance offering information on perioperative parameters were used.

**Results:** Despite the heterogeneity of studies, they support the view that innate immune response is activated to a greater degree in open colectomy (OC), which may be related to the more extensive trauma and surgical stress. On the other hand, cellular immunity is better preserved after LC. These differences are more pronounced in the immediate postoperative period.

**Conclusions:** LC has been related to decreased up-regulation of innate immunity and better-preserved cellular immunity. The latter may be related to better anti-tumor activity and may be beneficial in terms of oncological survival in a subgroup of LC patients.

## INTRODUCTION

Colorectal cancer (CRC) is the third most frequent type of cancer in western societies. The oncological equivalency of laparoscopic (LC) and open colectomy (OC) for cancer has already been proven at several randomized trials during the last decade [1, 2]. Shorter hospital stay, reduced postoperative pain, decreased intra-operative blood loss, better cosmesis, decreased wound infection rate and faster recovery are additional advantages of LC [3–6]. The Clinical Outcomes of Surgical Therapy Study (COST) Group reported that LC and OC for CRC provided comparable long-term outcomes [7]. Lacy *et al.* in their well-known ‘Barcelona trial’ found that LC was superior to OC in terms of morbidity, tumor recurrence and disease-free survival [8]. The authors suggested the presence of a significant survival advantage in favor of laparoscopic study arm for patients with stage III disease [8]. Interestingly, these results were attributed mainly to the better-preserved immunity in LC patients. This observation has shed light on the differences, between these two surgical approaches, in patients’ immunological status during the early postoperative period.

It is known that the amplitude of surgical trauma is directly related to the stimulation of the innate immune system in the surgical microenvironment and the secretion of a variety of cytokines, which stimulate a systemic response to stress [9]. It has been speculated—but not definitely proven—that the laparoscopic approach may attenuate this systemic innate immune response, and that may explain some of the short-term advantages of the LC [9]. During surgery, cancer cells are able to invade normal colorectal tissue, increasing the possibility of tumor recurrence. In particular, the level of circulating tumor cells is highest following manipulation of the tumor [10]. Cellular immunity may play a critical role in patients’ defense against cancer cells and the efficacy of this type of immunity during the surgery can be valuable in terms of disease-free survival [11]. Interestingly, recent data suggest that the innate response to surgical stress can inhibit the stimulation of cellular immunity during the early postoperative period [12]. One could speculate that if LC is really oncologically advantageous in the long term, it may be related to the decreased innate immune stress and better-preserved cellular immunity.

The aim of this review is to present data derived from interventional and prospective studies that focus on innate and cellular immunity after LC for cancer.

## SEARCH STRATEGY

This review is based on the results of bibliographic searches of PubMed, EMBASE, the Cochrane Library and Google Scholar. Searches of the literature up to December 2012, unrestricted by language, were performed applying combinations of the following terms: ‘laparoscopic colectomy’, ‘open colectomy’, ‘colorectal cancer’, ‘postoperative immune response’, ‘postoperative innate immune response’, ‘surgical stress’, ‘postoperative cellular immunity’, ‘cytokines’ and ‘inflammatory response’. In addition, we identified relevant trials from the reference list of each selected article. All studies published on ‘postoperative immune response’, ‘postoperative innate immune response’, ‘postoperative cellular immunity’, ‘surgical stress’ and ‘surgical stress laparoscopy versus open’ were collected and, from these, the ones that referred to CRC, laparoscopic and open colectomy and that had any clinical relevance were used for analysis in the present review. Exclusion criteria for the clinical studies were based on the type of study (e.g. review) and specific study population (e.g. patients with metastatic disease and radiation therapy). Because of the limited studies focused on colectomy for cancer, we selected a small number of trials focusing on other indications (e.g. inflammatory bowel disease and cholecystectomy) when there was a connection in terms of immunological mechanisms. When multiple articles for a single study were present, we used the latest publication and supplemented it with data from the previous publications. Neither publication status nor language of publication was an exclusion criterion for this review. All the clinical studies are presented in Tables 1 (innate immunity) and 2 (cellular immunity). When the indication is not colectomy for cancer it is specifically mentioned.

**Table 1:** Clinical studies included in the evaluation of innate immunity

Author; reference	Type of study	Number of patients (Total; Open; Lap.)	Examined cytokines/cells	Time-points	Main Results
Han *et al. *[17]	Prospective study	Total: 74; Open: 39, Lap: 35	CRP, WBC, ESR, CD4, CD8, CD4/CD8, HLA-DR	Pre-op, POD1, POD5	Higher HLADR in lap group in POD5 (*P = *0.015)
Wang *et al. *[18]	Prospective study	Total: 163; TOG:42, FTOG:41, TLG: 40, FTLG: 40	IL-6, CRP, CD3, CD4, CD8, CD4/CD8	Pre-op, POD1, POD3, POD5	Lower CRP and IL-6 in lap and FT groups (*P < *0.005) and higher CD4/CD8 in lap groups in all post-op time-points
Tsamis *et al.* [16]	Retrospective case – control matched study	Total: 66; Open: 30; Lap: 36	CRP, IL-1, IL-6, IL-8, INF-γ	Pre-op, POD1, POD7	Increased INF-γ in lap group in PDO1 and POD7 (*P = *0.001)
Harmon *et al.* [20]	Prospective study	Total: 53; Open: 41; Lap: 12	Cortisol, IL-1, IL-6	Pre-op, 1 h, 2 h, 3 h, 4 h, 5 h, 12 h, 24 h, 24 h post-op	IL-6 lower in lap group 2-24 h post-op (*P < *0.005)
Schwenk *et al.* [21]	Randomized controlled study	Total: 60; Open: 30; Lap: 30	IL-6, IL-6 Receptor antagonist, IL-10, CRP	Pre-op, 1 h and 4 h post-op and POD1, POD2, POD4, POD7	Post-op peak IL-6 (*P = *0.05) and CRP (*P < *0.001) and overall post-op IL-6 (*P = *0.03) and CRP (*P = *0.002) lower in the lap group
Braga *et al.* [22]	Randomized controlled study	Total: 79; Open: 39; Lap: 30	Cortisol, CRP, CD4, CD8, CD4/CD8	Pre-op, 24 h, 48 h and 72 h post-op and PDO7	Higher CD4/CD8 (P = 0.01) on POD1 and faster return of CRP to pre-op values (*P* = 0.01)
Delgado *et al.* [23]	Randomized prospective study	Total: 97; Open: 58; Lap: 39	cortisol, prolactin, CRP, IL-6	Pre-op, 4 h, 12 h, 24 h, 72 h post-op	IL-6 higher in open group at 4, 12 and 24 h post-op, CRP lower in laps at 72 h post-op
Dunker *et al.* [24]	Randomized controlled study	Total: 30; Open: 14; Lap: 16 Crohn's disease, ulcerative colitis and familial adenomatous polyposis	CRP, IL-6, HLA-DR	Pre-op, POD1 and POD7	No statistical significant difference. Higher CRP and IL-6 in open group
Leung *et al.* [25]	Prospective randomized study	Total: 34; Open: 17; Lap: 17	IL-1b, TNF-a, IL-6, CRP	Pre-op, 2 h, 8 h, 12 h, 48 h, 72 h, 1 week and 4 weeks post-op	Peaked IL-1b and IL-6 2 h CRP 48 h post-op decreased in laps
Tsimogiannis *et al.* [27]	Randomized controlled study	Total: 40; Open: 20; Lap: 20	a-defensins, IL-6, hs-CRP,	Pre-op, 5 min, 6 h and 24 h post-op	a-defensins lower in laps 5 min and 24 h post-op (*P < *0.002 and *P < *0.007), IL-6 lower in laps at 6 h and 24 h post-op (*P < *0.0001), hs-CRP lower in laps at 24 h post-op (*P < *0.001)
Tsimogiannis *et al.* [30]	Randomized controlled study	Total: 40; Open: 20; Lap: 20	TLR-2, TLR-4, IL-6, TNF-a, hs-CRP	Pre-op, 5 min, 6 h and 24 h post-op	IL-6 higher in open at 6 and 24 h post-op (*P < *0.0001), hs-CRP higher in open at 24 h post-op (*P < *0.001), TLR-2 higher in open at 5 min (*P = *0.013) and 24 h (*P = *0.007) post-op, TLR-4 higher in open at 5 min post-op (*P = *0.03)
Ozawa *et al.* [31]	Prospective study	Total 16; Open: 8; Lap: 8	Serum IL-6, IL-6 in ascites, ADH, ACTH, cortisol	Pre-op, 2 h, 4 h, 6 h, 8 h, 24 h, 48 h, 72 h post-op	IL-6 lower in laps at 4 h post-op (*P < *0.05), no statistically significant differences in IL-6 in ascites, ADH, ACTH or cortisol
Wu *et al.* [32]	Randomized prospective study	Total: 26; Open: 14; Lap: 12	IL-6, IL-8, TNF-a in serum and peritoneal fluid, WBC, CRP, HLA-DR	Pre-op, 2 h post-op, POD1, POD4	Serum IL-6 and IL-8 lower in laps at 2 h post-op (*P = *0.03) WBC and HLA-DR normalized earlier in laps (*P < *0.05)
Veenhof *et al.* [9]	Randomized prospective study	Total: 40; Open: 18; Lap: 22	WBC, CRP, IL-6, IL-8. HLA-DR, GH, Prolactin, cortisol	Pre-op, 2 h, 24 h, 72 h post-op	HLA-DR higher in laps (*P = *0.014), IL-6 lower in laps (*P = *0.003) at 2 h post-op

CRP = C-reactive protein, hs-CRP = high sensitivity CRP, WBC = White blood cells, IL = interleukin, HLA-DR = Humal leukocyte antigen DR, TNF-a = Tumor necrosis factor-a, ADH = antidiouretic hormone, ACTH = adrenocortical hormone, GH = growth hormone, TLR = Toll like receptor, INF-γ = interpherone γ, pre-op = pre-operatively, post-op = postoperatively, POD = postoperative day

**Table 2:** Clinical studies included in the evaluation of cellular immunity

Author; reference	Type of study	Number of patients (Total, Open, Lap.)	Examined cytokines/cells	Time-points	Main results
Decker *et al.* [35]	Prospective study	Total: 43; Open: 18; Lap: 25 Cholocystectomy for symptomatic cholelithiasis	Th1/Th2 balance, IL-4, TNF-a, CD30, CD23	Pre-op, 2, 24 and 48 post-op	Higher IL-4 and Th2 activity in open group post-op and higher Th1 activity in laps post-op
Huang *et al.*[36]	Prospective study	Total: 68; Open: 33; Lap: 35	WBC, Neutrophils, CD4, CD8, CD4/CD8, CD45RO^+^T, NKC, CRP	Pre-op, POD1, POD4, POD7	CD4 and CD8 higher in laps on POD4 (*P < *0.05)
Wang *et al.*[18]	Prospective study	Total: 163 (TOG:42, FTOG:41, TLG: 40, FTLG: 40)	IL-6, CRP, CD3, CD4, CD8, CD4/CD8	Pre-op, POD1, POD3, POD5	Lower CRP and IL-6 in lap and FT groups (*P < *0.005) and higher CD4/CD8 in lap groups in all post-op time-points
Fujii *et al.*[40]	Prospective study	Total: 20; Open: 10; Lap: 10 Distal gastrectomy for gastric cancer	WBC, CD3, CD4, CD8, CD57, HLA-DR-positive lymphocytes, INF-γ, IL-4	Pre-op, POD1, POD3 and POD7	INF-γ decreased in open group compared with laps in POD3 (*P < *0.05). IL-4 increased post-op only in open group
Whelan *et al.*[12]	Prospective study	Total: 35; Open: 17; Lap: 18	DHT challenge	Pre-op, immediately after surgery, POD3	Post-op challenges were significantly smaller than pre-op only in open group. The differences between groups were significant immediately after surgery and not in POD3
Han *et al.*[17]	Prospective study	Total: 74; Open: 39; Lap: 35	CRP, WBC, ESR, CD4, CD8, CD4/CD8, HLA-DR	Pre-op, POD1, POD5	Higher HLADR in lap group in POD5 (*P = *0.015)
Ordemann *et al.*[46]	Randomized prospective study	Total: 40; Open: 20; Open: 20	WBC, CD4, CD8, CD4/CD8, HLA-DR in CD14 monocytes. IL-6, TNF-a	Pre-op, POD1 to POD4	Post-op peak of WBC lower in laps (*P < *0.05) HLA-DR expression was lower in open group in POD4. Lower post-op levels of IL-6 and TNF-a in laps.
Veenhof *et al.*[9]	Randomized prospective study	Total: 40; Open: 18; Lap: 22	WBC, CRP, IL-6, IL-8. HLA-DR, GH, Prolactin, cortisol	Pre-op, 2 h, 24 h, 72 h post-op	HLA-DR higher in laps (*P = *0.014), IL-6 lower in laps (*P = *0.003) at 2 h post-op

WBC: White blood cells, NKC: natural killer cells, IL-6: interleukin 6, IL-8: interleukin 8, HLA-DR: Humal leukocyte antigen DR, TNF-a: Tumor necrosis factor-a, INF-γ: interpherone γ, DHT: delayed- type hypersensitivity, GH: growth hormone, pre-op: pre-operatively, post-op: postoperatively, POD: postoperative day

## INNATE IMMUNITY

Surgical interventions in the gastrointestinal track lead to activation of multiple mechanisms related to innate immunity. At the level of surgical microenvironment, macrophages are stimulated and secrete a variety of cytokines including interleukin (IL)-1 and tumor necrosis factor-a (TNF-a) which are directly correlated with the magnitude of surgical trauma [13, 14]. It is also known that IL-1 promotes the secretion of IL-6 from Kupffer cells in the liver, resulting in endocrine secretion of C-reactive protein (CRP) from liver cells, which finally stimulates a systematic stress response. The CRP values can be used to monitor the magnitude of surgical trauma as well [15]. IL-8 is another important cytokine, mainly secreted by macrophages and lymphocytes in the surgical microenvironment, acting as chemoattractant for neutrophils and enhancing the inflammatory response, while IL-10 is believed to inhibit the local and systemic inflammatory response [16]. The postoperative systemic response has also been evaluated by the levels of stress hormones including cortisol, prolactin and growth hormone, which may play a critical role in the down-regulation of cellular immunity [9].

Consistent with the pathophysiology of early postoperative stress, numerous recent reports suggest an increase of stress cytokines early during the postoperative period following colectomy for cancer. However, the comparison between LC and OC may be much more important in terms of evaluation of the innate immune response. Han *et al.* showed that white blood cells (WBC), CRP and erythrocyte sedimentation rate (ESR) were significantly elevated during the first five postoperative days (PODs), compared with pre-operative values, but they did not find any differences between the LC and OC groups [17]. Wang *et al.*, in their report evaluating traditional and fast track approaches, suggest a significant increase of IL-6 and CRP during the first five PODs in both LC and OC groups, but they found that the values of postoperative CRP and IL-6 in the traditional laparoscopic operation group and fast-track laparoscopic operation group were significantly lower, compared with the open groups [18]. Tsamis *et al.* showed that IL-6 and CRP were significantly higher during the first seven PODs, compared with baseline, in both LC and OC but the values were significantly higher in the first 24 hours compared with the seventh POD. Moreover, they could not find any differences between the two groups in terms of innate immune response [19]. Harmon *et al.*, in an early study, suggested that serum IL-6 was significantly lower in a laparoscopic group (12 patients) compared with an open group (41 patients) between 3 and 24 hours postoperatively [20]. Finally, Schwenk *et al.*, in 30 laparoscopic and 30 open colorectal resections for cancer, found that postoperative CRP and IL-6 values were significantly lower in the laparoscopic than in the open group [21].

According to Braga *et al.*, who evaluated the CRP levels of 40 patients who underwent laparoscopic colectomy and 39 patients who underwent open colectomy, CRP returned faster to pre-operative values (*P = *0.01) in the laparoscopic group, compared with the open colorectal resection group [22]. Delgado *et al.*, in a randomized clinical study including 58 patients submitted to open colectomy and 39 undergoing laparoscopic-assisted colectomy, showed that the levels of interleukin-6 were significantly higher at 4, 12 and 24 hours after surgery in the patients undergoing open colectomy, while the CRP levels were significantly lower at 72 hours after surgery in patients receiving laparoscopic-assisted colectomy [23]. Dunker *et al.* showed that CRP is significantly higher only on the first postoperative day after open colectomy for cancer and that, later, there are no differences between the two groups, while there are no differences at all in terms of IL-6 [24]. Finally, Leung *et al.* included 34 patients with rectosigmoid carcinoma without evidence of metastatic disease, and conducted a randomized clinical trial with 17 patients undergoing LC and 17 OC. They evaluated the serum levels of IL-6, IL-1b, TNF-a and CRP before and after surgery. They showed that both IL-6 and IL-1b peaked 2 hours after surgery, while the responses were significantly lower in laparoscopic- when compared with the open surgery group. On the other hand, there were no differences in terms of TNF-a values before and after surgery and between experimental groups [25].

Additional, less-known cytokines have already been used as markers of innate immune responses after colectomy for CRC. Tsimogiannis *et al.* used a-defensins, which are contained in Paneth cells and are effective against various micro-organisms as early markers of innate immune stimulation [26]. They conducted a randomized clinical trial, including 20 patients who underwent OC and 20 patients who underwent LC for CRC. They showed that a-defensins were lower 5 minutes and 24 hours after surgery in the laparoscopic group, compared with open group (*P < *0.002 and *P < *0.007 respectively). Interestingly, they found that IL-6 and CRP were lower in the same group of patients 6- and 24 hours after surgery, confirming the data presented in previous studies [27]. Toll-like receptors (TLRs) are believed to serve as the pattern recognition receptor in the innate immune defense system [28]. TLRs’ expression and serum levels have been related to surgical stress and innate immune stimulation [29]. The same group of authors evaluated the serum levels of TLR2, TLR4, TNF-a, IL-6 and high-sensitivity CRP (hsCRP) 5 minutes after deflation of the pneumoperitoneum in patients undergoing laparoscopic colectomy, 5 minutes after division of the colon in patients undergoing open colectomy and at 6 and 24 hours after surgery. They found that the levels of IL-6 were significantly higher in the open group 6- and 24 hours postoperatively and the hsCRP levels were significantly higher only at 24 hours after surgery. Consistent with previous studies, TNF-a did not differ between the two groups, while TLR2 levels were significantly higher in the open group 5 minutes and 24 hours after surgery. Finally TLR4 was higher only 5 minutes after surgery in patients who underwent open colectomy [30]. These data further support the idea that LC decreases the stimulation of innate immune system.

Differences between LC and OC in terms of innate immune response seem to be more obvious in the first postoperative hours. Ozawa *et al.* supported that serum IL-6 levels were significantly lower in the LC group 4 hours after surgery while IL-6 levels in the collected ascites samples were not significantly different between the two groups [31]. Wu *et al.* suggested that CRP values were comparable in the LC and OC groups, while IL-6 levels were significantly higher in the OC group only in the first 2 hours after surgery. They did not find any difference in the levels of inflammatory cytokines in the peritoneal drain fluid samples in the two groups. In the same study, LC patients were found to have lower serum levels of IL-8 two hours after LC compared with OC patients [32]. Consistent with the above data, Veenhof *et al.*, in a recent report, found significantly lower elevation of IL-6 in the LC group 2 hours after surgery while, after this time-point, the values were comparable between the two groups. CRP and IL-8 were comparable from the beginning of the evaluation until 72 hours after surgery. The authors could not demonstrate a difference between the two groups regarding the values of cortisol, growth hormone and prolactin during the same period of time [9].

In conclusion, there is much evidence that the innate immune response is less severe under the laparoscopic approach during the first hours after surgery for CRC. IL-6 and CRP are both markers of systemic inflammatory response and their early serum values are very sensitive indicators of the surgical stress, but IL-6 seems to better reflect the difference between the two approaches. It should be mentioned, though, that the heterogeneity of studies, the variety of cytokines being implicated in different pathways of the innate immunity and, finally, the different assessment time-points in different studies render the attainment of definitive conclusions really challenging.

## CELLULAR IMMUNITY

Tumor immunity, mainly defined as the cellular immune responses in the body against tumor, is believed to be critical for defense against cancer cells, especially during surgical interventions in CRC patients [33, 34]. The cellular immunity is mainly related to the function of antigen-presenting cells, including macrophages and dendritic cells which present tumor antigens and stimulate Th1 cells. The cell- mediated immune response includes the secretion of cytokines such as IFN-γ, IL-2, IL-12 and TNF-β from Th1 cells, the activation of macrophages and the induction of cytotoxic cells against cancer cells. Cellular immunity involves the stimulation of CD4+ and CD8+ T cells, while the counts of these cells and the CD4/CD8 ratio can be a sensitive marker of cellular immunity preservation [35]. In addition, mononuclear cells, which are known antigen-presenting cells, can be essential for the identification of tumor pathogens and the initiation of specific immune responses against cancer cells. The class II major histocompatibility (MHC-II) molecules and specifically human leukocyte antigen DR (HLA-DR), expressed on the surface of monocytes and macrophages, are essential for mediating the antigen presentation for specific immune response in humans. These class II MHC molecules in monocytes are up-regulated by Th1 cytokines and have been shown to be decreased after tissue trauma [12]. The expression of HLA-DR in the surface of antigen-presenting cells is critical for the activation of naïve CD4+ T cells and their further regulation and differentiation to Th1 and Th2 cells, highlighting the important role of these molecules in the preservation of cellular immunity. It should be mentioned that HLA-DR is not implicated directly in the activation of CD8+ T cells. Moreover INF-γ secreted by Th1 could enhance the activity of natural killer cells (NKC) which can fight against cancer cells through antibody-dependent, cell-mediated cytotoxicity. Finally, part of activated T cells can be differentiated into memory T cells (CD45RO+ T cells), which have a more rapid and severe reaction against tumor cells and twice the degree of antitumor immune responses [11].

### Cells and cytokines

Overall, based on recent data from prospective clinical studies, surgical stress and up-regulation of innate immunity inhibits the stimulation of cellular immune response. In particular, Decker *et al.*, in a study evaluating laparoscopic versus open cholecystectomy for symptomatic cholelithiasis, have shown that cell-mediated immunity is down-regulated while antibody-mediated immunity is up-regulated after every surgery, which seems to be obvious via a shift in the Th1/Th2 balance toward Th2 two hours after surgical incision [35]. Huang *et al.* showed that CD4+, CD8+ and NK cell counts were decreased in both groups after surgery but, in the LC group, the CD4+, CD8+ and CD45RO+ counts were significantly higher, compared with the OC group on the fourth POD whilst, in the first and fifth, there was no significant difference. Interestingly, the count of NK cells in the OC group had a continuous depression trend compared with the LC group. Finally, the count of CD4+, CD8+ and CD45RO+ returned to pre-operative levels on the seventh POD in the LC group, while only the counts of CD4+ and CD45RO+ returned to pre-operative levels in the OC group, suggesting a continuous depression of CD8+ cells, which are critical for the anti-cancer effect of cellular immunity [36].

However, Wang *et al.*, in a recent report comparing traditional and fast track approaches, found that the count of CD3+ cells is decreased immediately after surgery in LC and OC, but the levels of CD3+ cells were higher in the LC group compared with OC in both traditional and fast track approach on the first, third and fifth PODs. In the same study, the authors suggest that the count of CD4+ cells is lower in the traditional open group, compared with the other groups (fast track open, fast track laparoscopic and traditional laparoscopic) during the first three PODs, contrary to Huang’s previous data. They also mentioned that the ratio of CD4+/CD8+ is decreased after surgery in all four groups, but had a more significant reduction in the traditional open group, compared with the traditional laparoscopic group, during the first three PODs, while there was no significant difference in the counts of CD8+ cells, before and after surgery, in each group during the evaluated postoperative period [18]. The confounding results in those two studies may reflect the different distribution of samples in terms of cancer location. In particular, Huang *et al.* included more samples from rectal cancers [36].

Berguer *et al.*, in an early study using a rat animal model, evaluated the expression of IL-2 receptor (IL-2R) in CD4+ and CD8+ cells and the levels of corticosterone 24 hours after open- and laparoscopic fundoplication. They showed that IL-2R expression in CD4+ cells was significantly higher and serum corticosterone was significantly lower in the laparoscopic, compared with the open group, while there was no difference in the IL-2R expression in CD8+ cells [37]. These data support the main hypothesis that, during laparoscopic surgery, the decreased surgical stress permits the better preservation of cellular immunity in the early postoperative period.

As mentioned above, IFN-γ, which is a cytokine secreted by Th1 cells, can activate macrophages and NK cells and is believed to be one of the principal effectors of cell-mediated immunity and delayed-type hypersensitivity reaction [38]. On the other hand, Th2 cells produce IL-4, IL-5 and IL-10, which activate antibody production by B cells and suppress cell-mediated immunity [38]. Recent reports support the view that higher levels of INF-γ may indicate better-preserved cell-mediated immune function [36, 39]. Fujii *et al.*, in a study focusing on distal gastrectomy for gastric cancer, showed that laparoscopic procedure is followed by increased INF-γ but stable IL-4, compared with the open group, where there is significant decrease of INF-γ and increase of IL-4, suggesting that the laparoscopic approach may be related to better Th1 function, while the open approach clearly leads to activation of Th2 mediated immunity and B cell stimulation [40]. Decker *et al.* have also shown that the down-regulation of INF-γ is less significant in laparoscopic procedures, compared with open procedures, while the Th2-secreted cytokines—such as IL-4—are less down-regulated in open procedures in the two-hour period after surgery [35]. These observations were also strengthened by Livingston *et al.*, who proved that IFN-γ and IL-2 productions were depressed after severe injury. Specifically, they measured mitogen-stimulated INF-γ production sequentially on days 1, 3, 7, 14 and 21 after admission in 20 multiply injured patients. Ten patients recovered uneventfully and ten developed a major infection, three of them dying. Trauma resulted in immediate and profound depression of INF-γ production compared with controls, which lasted 21 days in uninfected patients, while failure to increase the INF-γ levels back to normal was related to an episode of major infection [41]. Interestingly, it has already been shown that LC demonstrated better preservation of the cellular immunity but not the humoral immunity [42]. These data regarding inflammatory cytokines suggest that laparoscopy may inhibit the stress- induced Th1/Th2 shift after surgery, probably through decreased surgical injury and stress.

Finally, as mentioned above, delayed-type hypersensitivity (DTH) responses are associated with T cell-related immunological function and have been used as markers of cell-mediated immunity by several studies—mainly in animal models. Bessler *et al.* used a DHT-type model in pigs, in order to examine the difference in T- cell function after LC and OC, and have concluded that DTH response is better maintained in laparoscopic—compared with open—procedures, supporting the hypothesis of the better-preserved cell-mediated immune response after laparoscopic approaches [43]. Skin tests showed that the group of animals that underwent laparotomy had significantly diminished responses to keyhole limpet hemocyanine (KLH) when challenged postoperatively, despite having normal responses pre-operatively. Finally, Whelan *et al.* evaluated DTH challenges at three time points in patients who underwent laparoscopic or open colorectal excision: pre-operatively, immediately following surgery and on the third postoperative day (POD 3). They showed that postoperative DTH responses were significantly weaker in patients after open surgery, compared with laparoscopic groups [12].

### The role of HLA

As presented above, the role of HLA class II, including HLA-DR, is pivotal in the cellular immune system. If these antigens are expressed in small amounts on monocytes, they abort the action of tumor-specific T cells and they fail to develop into functional dendritic cells, leading to deficit of antigen-presenting cells, which are responsible for the stimulation of naïve CD4+ T cells. This mechanism has been thoroughly analysed by Wang *et al.* for myeloma and by Kusmartsev *et al.* in their review focusing on immature myeloid cells and cancer-associated immune suppression [18, 44]. The HLA class II monocytes are converted into functionally mature macrophages, an activity which is impaired if there is lower expression of HLA. The reduction of functional macrophages negates the immunity of the cells that infiltrate the site of primary T cell-mediated killing, digest the resulting residues and represent antigens from the smashed cells. This failing procedure affects the duration and propagation of T cell attack on tumor cells. This is the way that the deficit of the cell-mediated response after an open conventional colectomy correlates to impairment of the cancer surveillance system. Besides, De Bruin *et al.* have supported the view that the higher epithelial HLA-DR expression predicts reduced recurrence rates locally and distantly, and prolonged survival in rectal cancer patients: a suggestion that confirms the above-mentioned [45]. On the other hand, according to Vuk-Pavlovic *et al.*, HLA-DR expression, showing little variation with age, gender or race, provides a reliable marker of the relative immune capacity of the host. It may therefore be worthwhile to quantify the role of tumor antigen re-presentation in clinical efficacy of therapeutic vaccination against tumors [33].

Based on the data presented above, HLAs can be very sensitive markers of cellular immune activation. Han *et al.*, in a recent prospective non-randomized clinical study focused only on patients with stage III disease, have shown that the LC group had a better-preserved mHLA-DR on the fifth postoperative day, compared with OC (*P < *0.014), and faster recovery. The total lymphocyte counts, CD4+ T cell counts, CD8+ T cell counts and the CD4/8 T cell ratio decreased equally after surgery in both the LC and OC groups (17). According to a randomized control trial by Ordemann *et al.*, the mHLA-DR expression examined 1 h before surgery and 6 h, 12 h and 24 h postoperatively, was found to be preserved in LC, in contrast to OC [46]. Finally, Veenhof *et al.*, in a prospective, randomized trial evaluating the immune response in patients after open (18 patients) and laparoscopic (22 patients) surgery for rectal cancer, showed that the LC group presented higher expression of HLA-DR two hours after surgery, compared with the OC group (*P = *0.015) [9]. Consequently, these data demonstrate a cell-mediated immunological benefit and an earlier immunological recovery, expressed mainly by HLA-DR expression with the LC.

## CONCLUSION

The oncological equivalency of laparoscopic colectomy for cancer with its open counterpart has been well established in randomized trials during the last decade. Data from an isolated report, which need to be confirmed in other studies, suggested that the laparoscopic approach may provide a long-term advantage in terms of disease-free survival, especially for patients with stage III disease. According to the latest reports, systemic innate immune response, as expressed by secretion of CRP, IL6 and other innate cytokines, is decreased in the laparoscopic approach, probably due to decreased surgical stress. The results remain controversial but the majority of trials show a benefit in favor of laparoscopic colectomy in terms of innate immune response, especially in the first postoperative hours. Cellular immunity seems to be less down-regulated in LC, compared with OC. In particular, despite the presence of conflicting results in some studies, T cell counts, INF-γ, IL-2 and HLA-DR are less decreased after laparoscopic surgery, suggesting better-preserved cellular immunity after LC. One could explain these results by the concept that increased activation of innate immunity results in more severe inhibition of cellular immune mechanisms. Finally, the latest data suggest a decreased Th1/Th2 shift during the early post-LC period, which probably also leads to better-preserved cellular immunity in patients managed with LC for their CRC. It should be mentioned, though, that the results from the clinical trials are not yet definitive. This can be explained by the significant diversity in terms of the numbers of patients, the different time-points and the different markers of immunity used for evaluation. Further multi-center, randomized, clinical trials, using the same basic markers for cellular and innate immunity, are needed to establish this possible difference in immune response and to determine the importance of this difference in patients’ oncological outcomes. Focusing on inter-relations between innate immunity activation and cellular immunity depression after LC, compared with OC, may also shed light on the postoperative immunological occurrences that have been studied extensively—but still not thoroughly or in depth.

**Conflict of interest:** none declared
